# Enhanced antibacterial effect of the novel T4-like bacteriophage KARL-1 in combination with antibiotics against multi-drug resistant *Acinetobacter baumannii*

**DOI:** 10.1038/s41598-018-32344-y

**Published:** 2018-09-20

**Authors:** Mathias Jansen, Adam Wahida, Simone Latz, Alex Krüttgen, Helga Häfner, Eva Miriam Buhl, Klaus Ritter, Hans-Peter Horz

**Affiliations:** 1 Institute of Medical Microbiology, RWTH Aachen University Hospital, Aachen, Germany; 2 Third Medical Department for Haematology and Oncology, Klinikum rechts der Isar, Technical University of Munich, 81675 Munich, Germany; 3 Department of Infection Control and Infectious Diseases, RWTH Aachen University Hospital, Aachen, Germany; 4 Electron Microscopy Facility, Institute of Pathology, RWTH Aachen University Hospital, Aachen, Germany

## Abstract

The continuing rise of infections caused by multi-drug resistant bacteria has led to a renewed interest in bacteriophage therapy. Here we characterize phage vB_AbaM-KARL-1 with lytic activity against multi-drug resistant clinical isolates of *Acinetobacter baumannii* (AB). Besides genomic and phenotypic phage analysis, the objective of our study was to investigate the antibacterial outcome when the phage acts in concert with distinct antibiotics. KARL-1 belongs to the family of *Myoviridae* and is able to lyse 8 of 20 (40%) tested clinical isolates. Its double-stranded DNA genome consists of 166,560 bp encoding for 253 open reading frames. Genome wide comparison suggests that KARL-1 is a novel species within the subfamily *Tevenvirinae*, sharing 77% nucleotide identity (coverage 58%) with phage ZZ1. The antibacterial efficacy at various multiplicities of infection (MOI) was monitored either alone or in combination with meropenem, ciprofloxacin, and colistin. A complete clearance of liquid cultures was achieved with KARL-1 at an MOI of 10^−1^ and meropenem (>128 mg/l). KARL-1 was still effective at an MOI of 10^−7^, but antibacterial activity was significantly augmented with meropenem. While ciprofloxacin did generally not support phage activity, the application of KARL-1 at an MOI of 10^−7^ and therapeutic doses of colistin significantly elevated bacterial suppression. Hence, KARL-1 represents a novel candidate for use against multi-drug resistant AB and the therapeutic outcome may be positively influenced by the addition of traditional antibiotics.

## Introduction

As multi-drug resistant bacteria are inexorably on the rise, alternative antibacterial strategies are urgently needed^1^. One option could be bacteriophage therapy, which is the use of viruses against bacteria, a concept that precedes the introduction of antibiotic drugs and which still finds consideration in countries of the former Soviet Union^2–4^. The bulk of infections associated with antibiotic-resistance are caused by members of the so called “ESKAPE” group, which consists *of Enterococcus faecium* and *Staphylococcus aureus* as well as gram-negative *Klebsiella pneumoniae*, *Acinetobacter baumannii*, *Pseudomonas aeruginosa*, and *Enterobacter* species^5,6^.

The species *A*. *baumannii* is of particular concern, as it has rapidly emerged in healthcare settings over the last years and is responsible for a number of outbreaks worldwide^7^. Its spectrum of infections is large and includes among others bacteremia, pneumonia, meningitis, urinary tract- and wound infections^8^. Being equipped with intrinsic and acquired antibiotic resistance mechanisms along with the capacity to withstand prolonged exposure to disinfectants, detergents, UV, and desiccation, makes the control of *A*. *baumannii* extremely challenging^8–10^.

For that reason, a number of *Acinetobacter* phages have been isolated and characterized in recent years and their principle suitability for therapeutic purposes has been explored based on *in vitro* and *in vivo* studies^11–22^.

Up to date there have been isolated around 100 *Acinetobacter* phages from different geographic regions of which the genomes of 37 *Acinetobacter* phages have recently been investigated in detail^23^. This work grouped the *Acinetobacter* phages in several distinct clusters, spanning all three families of the *Caudovirales* and represents a valuable framework for understanding the diversity and evolutionary relationship of *Acinetobacter* phages^23^. One bottom line of this study is the conclusion that our knowledge about the genetic diversity and biological versatility of *Acinetobacter* phages is merely a glimpse, and that the discovery and characterization of novel phages from different environments will continue to be of vital importance^23^. Here we characterize phage KARL-1 (vB_AbaM-KARL-1), isolated from an aquatic system, with lytic activity against topical multi-drug resistant clinical isolates of mostly carbapenemase-producing *A*. *baumannii* strains. Besides genomic and phenotypic characterization of this novel phage our particular interest was to study the antibacterial effects of KARL-1 alone and in combination with three unrelated antibiotics, i.e. meropenem, ciprofloxacin, and colistin. Positive interactions between antibiotics and phages have been shown for some bacterial species, e.g. *Escherichia coli*^24–26^, *Burkholderia cepacia*^27^, *Pseudomonas fluorescens*^28^ and also for some members of the ESKAPE group, e.g. for *S*. *aureus*^29,30^, *K*. *pneumoniae*^31^, and mostly for *P*. *aeruginosa*^32–40^. However, to the best of our knowledge this is the first study reporting an enhanced bacterial suppression with the combination of phage and antibiotics against multi-drug resistant clinical isolates of *A*. *baumannii*. The results of this study highlight the complementary nature of phage and antibiotic therapy when administered together against this nosocomial pathogen.

## Results

### Morphology and host range

**Figure 1:**
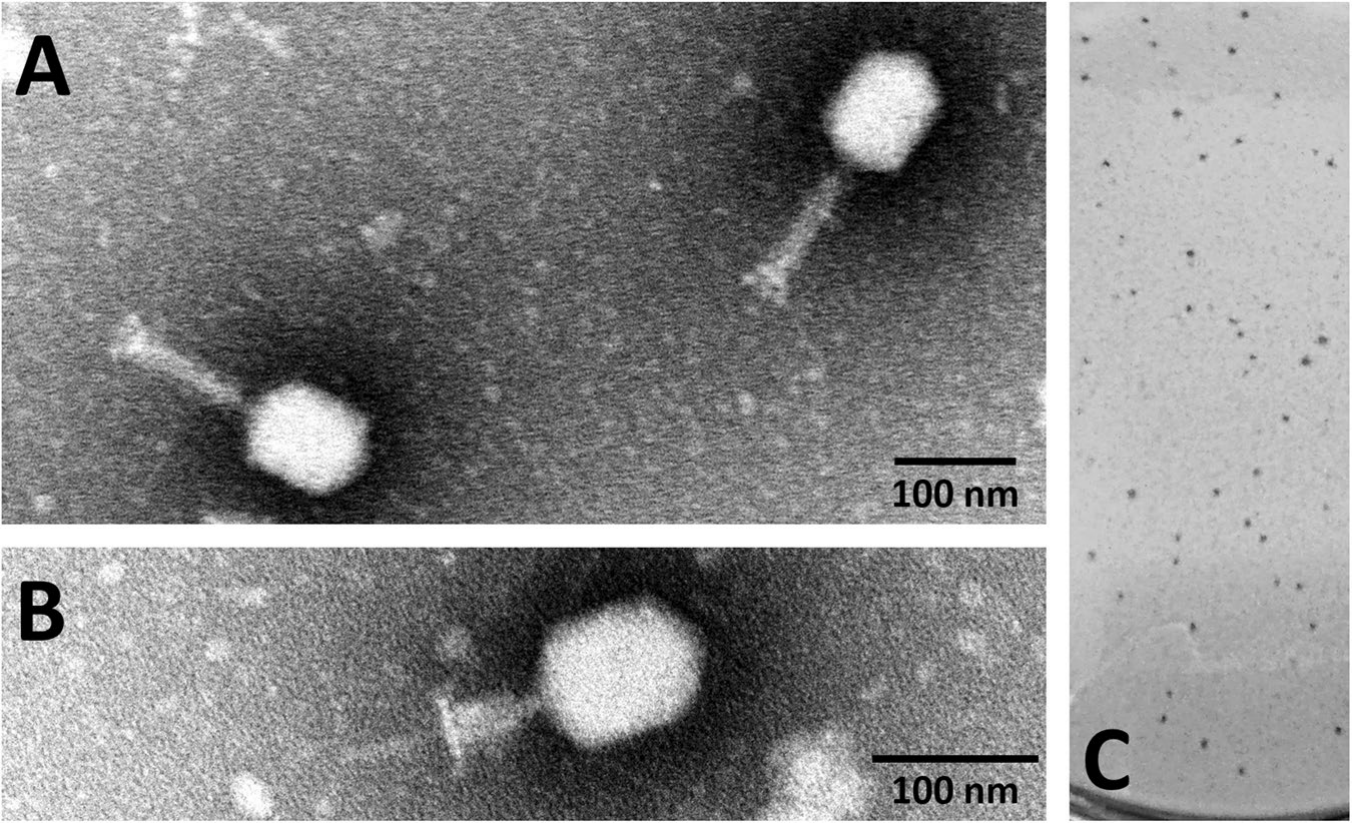
Electron micrograph images and plaque formation of KARL-1. (**A**) Phage with relaxed tail negatively stained with 1% phosphotungstic acid. (**B**) Phage with contracted tail and same staining method. (**C**) Phage plaques formed on the bacterial lawn of *A*. *baumannii* strain AB01.

KARL-1 was isolated from pond water based on the multi-drug resistant clinical isolate of *A*. *baumanni* “AB01”, as described previously^41^. Transmission electron microscopy (TEM) revealed that KARL-1 has the typical morphology of *Myoviridae* with an elongated icosahedral head (width 81.4 ± 1 nm; length 108.8 ± 1.7 nm), corresponding to Bradley morphology A2. In its relaxed form, the tail has a length of 113.8 ± 1.1 nm, whereas the contracted tail has a length of 47.3 ± 0.8 nm (Fig. 1A,B). On solid media KARL-1 produces relatively small plaques with a size of up to 0.8 mm in diameter without halos on the lawn of AB01 after incubation at 37 °C (Fig. 1C).

**Figure 2:**
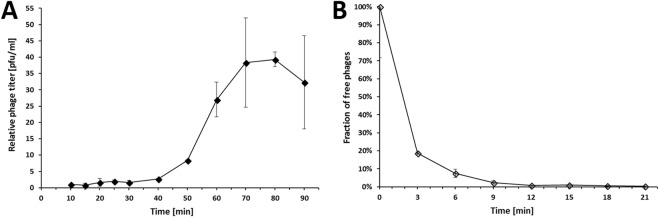
Phage characterization. (**A**) One-step growth curve of KARL-1 infecting *A*. *baumannii* clinical isolate AB01 at 37 °C. (**B**) Adsorption profile of KARL-1. Error bars denote standard deviation.

One-step growth experiments indicated a latent period of 30 min and a burst size of 39 ± 4 phage particles (Fig. 2A). The establishment of an adsorption profile showed that after 12 min more than 99% of phage particles had adsorbed to the host cells (Fig. 2B).

Lysis capacity of KARL-1 was observed for all tested temperatures, ranging from room temperature (~25 °C) to 45 °C. However, optimal temperature for lysis was 37 °C, see Supplemental Fig. S1.

For determination of the host range 20 multi-drug resistant clinical isolates, distinguishable by antibiotic resistance profiles, type of identified carbapenemases, and by genomic fingerprinting (see Supplementary Fig. S2), were tested along with seven reference strains. KARL-1 formed zones of clearing against 16/20 clinical isolates (80%) and against 5/7 reference strains (71%) when spotted undiluted (approximately 10^8^ pfu/ml). Upon further titration of phages continued lysis and plaque formation (indicative of productive phage infection) was observed for 8/20 clinical isolates (40%) and 2/7 reference strains (29%), respectively.

### Genome analysis

The genome of KARL-1 has a size of 166,560 bp and a GC-content of 36.79%. In total, 253 open readings frames (ORFs) were identified, varying in size from 31 to 1306 amino acid residues. Aside from seven tRNA genes (i.e. Met, Cys, Leu, Thr, Ser, Phe, Trp), 69 ORFs (27.3%) encode for proteins involved in replication, maturation, and release of phage progenies, 36 ORFs (14.2%) encode for structural phage proteins, 115 ORFs (45.5%) encode for hypothetical phage-like proteins, while the remaining 33 ORFs (13%) represent hypothetical proteins with no recognized phage origin. The full list of the 253 ORFs as well as their orientation of transcription can be found online as Supplementary Table S1 and Fig. S3, respectively. Genes encoding for an integrase, attachment sites, or virulence genes were not identified, likely indicating KARL-1 as obligatory lytic and as a potentially safe candidate for therapy against *A*. *baumannii*-associated infections. Comparison with publicly available phage genomes (Nucleotide BLAST) revealed closest relationship with recognized members of the subfamily *Tevenvirinae*, e.g. *Acinetobacter* phage ZZ1, with only moderate genome-wide nucleotide sequence similarity (Table 1, Fig. S4).

**Table 1:** Genome wide nucleotide identity of KARL-1 with other *Acinetobacter* phages.

Related phages	# ORFs	Query cover	Identity	Accession-no
*Acinetobacter* phage ZZ1	256	58%	77%	HQ698922.4
*Acinetobacter* phage Acj9	253	54%	71%	HM004124.1
*Acinetobacter* phage Acj61	241	50%	72%	GU911519.1
*Acinetobacter* phage Ac42	255	34%	69%	HM032710.1
*Acinetobacter* phage 133	257	25%	75%	HM114315.1

**Figure 3:**
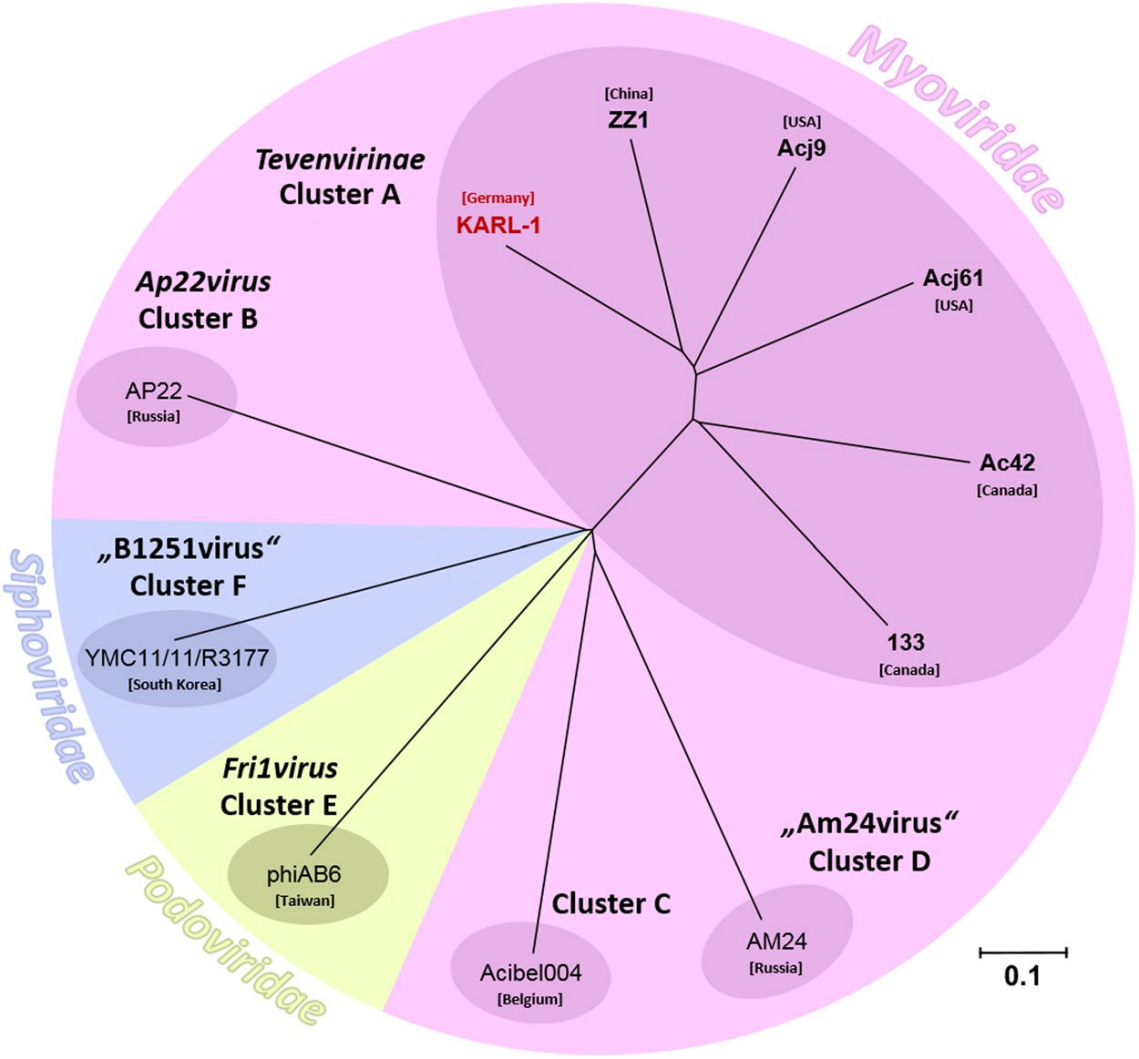
Phylogenetic analysis. Distance dendrogram showing the genetic relatedness of KARL-1 within the subfamily *Tevenvirinae* (Cluster A) and with representative members of other recognized clusters of *Acinetobacter* phages. The node labels represent individual phage genomes and their country of isolation. The accession numbers of the phage genomes of Clusters B-F are: AP22 (HE806280), Acibel004 (KJ473422), AM24 (KY000079), phiAB6 (KT339321), YMC11/11/R3177 (KP861230), for Cluster A: see Table 1. The scale bar represents the number of gene differences (presence or absence).

A neighbor joining tree based on pairwise Jaccard distances of shared versus separate (non-shared) genes grouped KARL-1 as a distinct lineage within the subfamily *Tevenvirinae* to the exclusion of other recognized *Acinetobacter* phage clusters, as described previously^23^, Fig. 3.

Altogether, KARL-1 and the five other members of Cluster A encode for a total of 655 different genes. Of these, there is a set of 111 single-copy core genes present in all six phages (Supplementary Table S1). Conversely, within the genome of KARL-1 there are 54 genes that are not present in the other phages (singletons). The number of singletons found in the other five phage species is of similar magnitude, ranging from 54 to 91 singletons.

**Figure 4:**
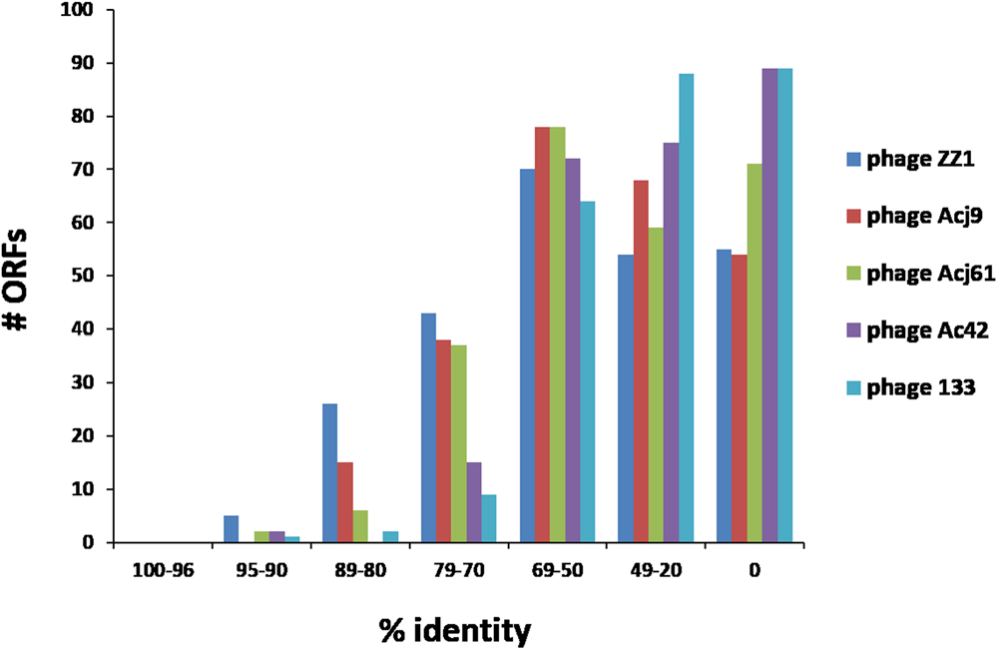
Comparison at protein level. Sequence identity of translated ORFs of KARL-1 with the five recognized members of the *Tevenvirinae* subfamily.

KARL-1 and ZZ1 share 5 ORFs with 90–95% sequence identity, 26 ORFs with 80–89% sequence identity, 43 ORFs with 70–79% sequence identity, and 70 ORFs with 50–69% sequence identity, Fig. 4. The remaining 109 ORFs of KARL-1 have sequence identities below 50% to those of phage ZZ1. Notably, there is no ORF in the genome of KARL-1 with sequence identities beyond 95% to any ORF from the five known phages within the *Tevenvirinae* subfamily (Fig. 4).

### Liquid infection assays

**Figure 5:**
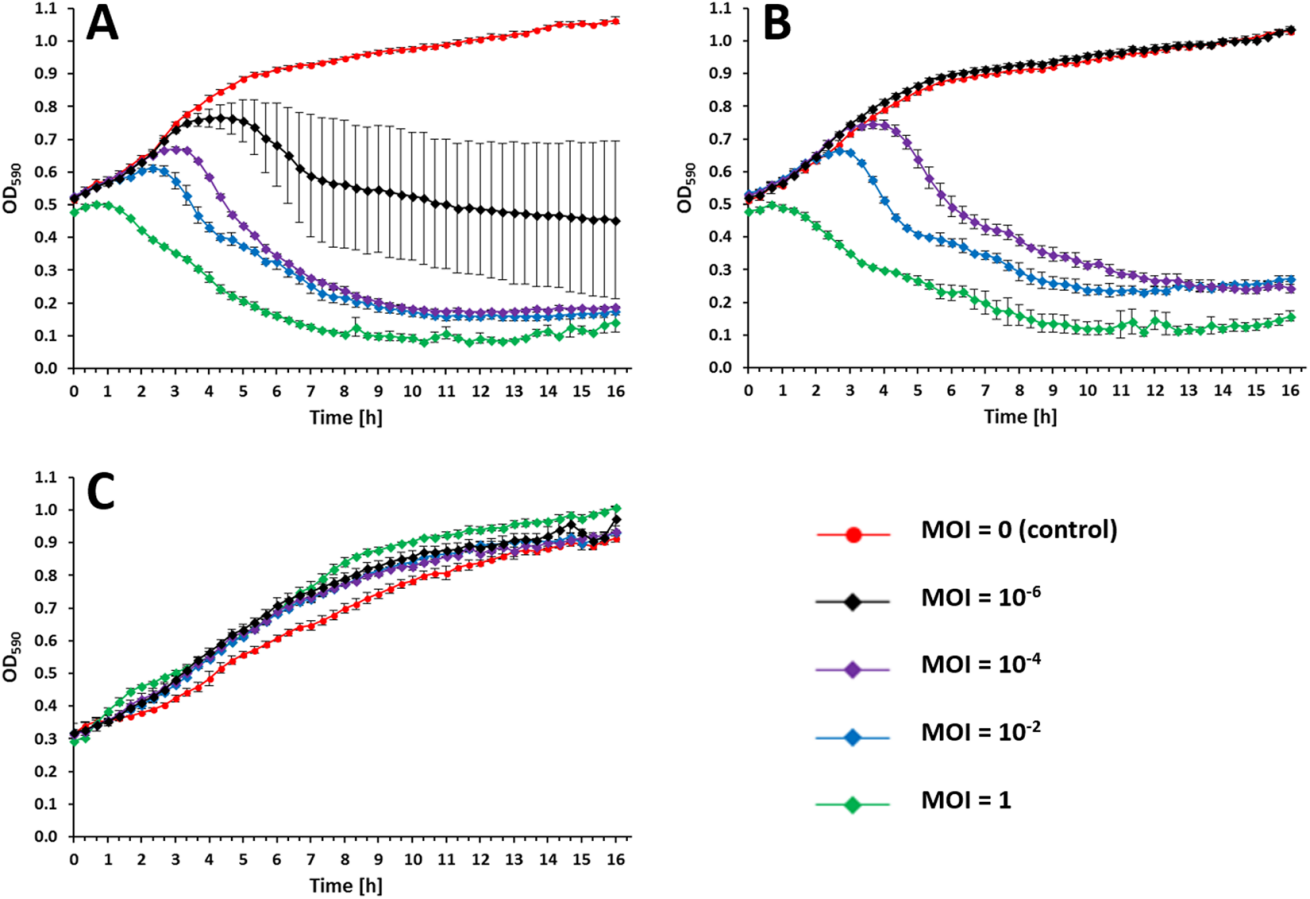
Growth/kill curves. Activity of KARL-1 against planktonic cells of the three different multi-drug resistant *A*. *baumannii* strains (**A**) AB01, (**B**) AB02, (**C**) AB03. Bacterial reduction was measured via optical density at 590 nm (OD_590_). Each experiment was performed in triplicate, bars indicate standard deviation.

The efficacy of KARL-1 against three selected *A*. *baumannii* strains (AB01-AB03) grown in liquid medium within a time frame of 16 h was assessed with four different multiplicities of infections (MOIs). KARL-1 was able to suppress the growth of its initial propagation strain AB01 at all MOIs. A reduction of the optical density (OD) at 590 nm from around 0.5 down to around 0.1 was achieved with MOIs 1 to 10^−4^ (Fig. 5A). Using the phage at an MOI of 10^−6^ caused a bacterial decline at about 4.5 hours of initial bacterial growth and returned the OD below the initial value (Fig. 5A). KARL-1 suppressed strain AB02 in a likewise manner, except for the lowest tested MOI 10^−6^, which was not effective (Fig. 5B). No suppressive effect was seen with the strain AB03, as a result of natural resistance against the phage, which is in agreement with the failure of the phage to produce plaques on solid media using the same strain (Fig. 5C).

**Figure 6:**
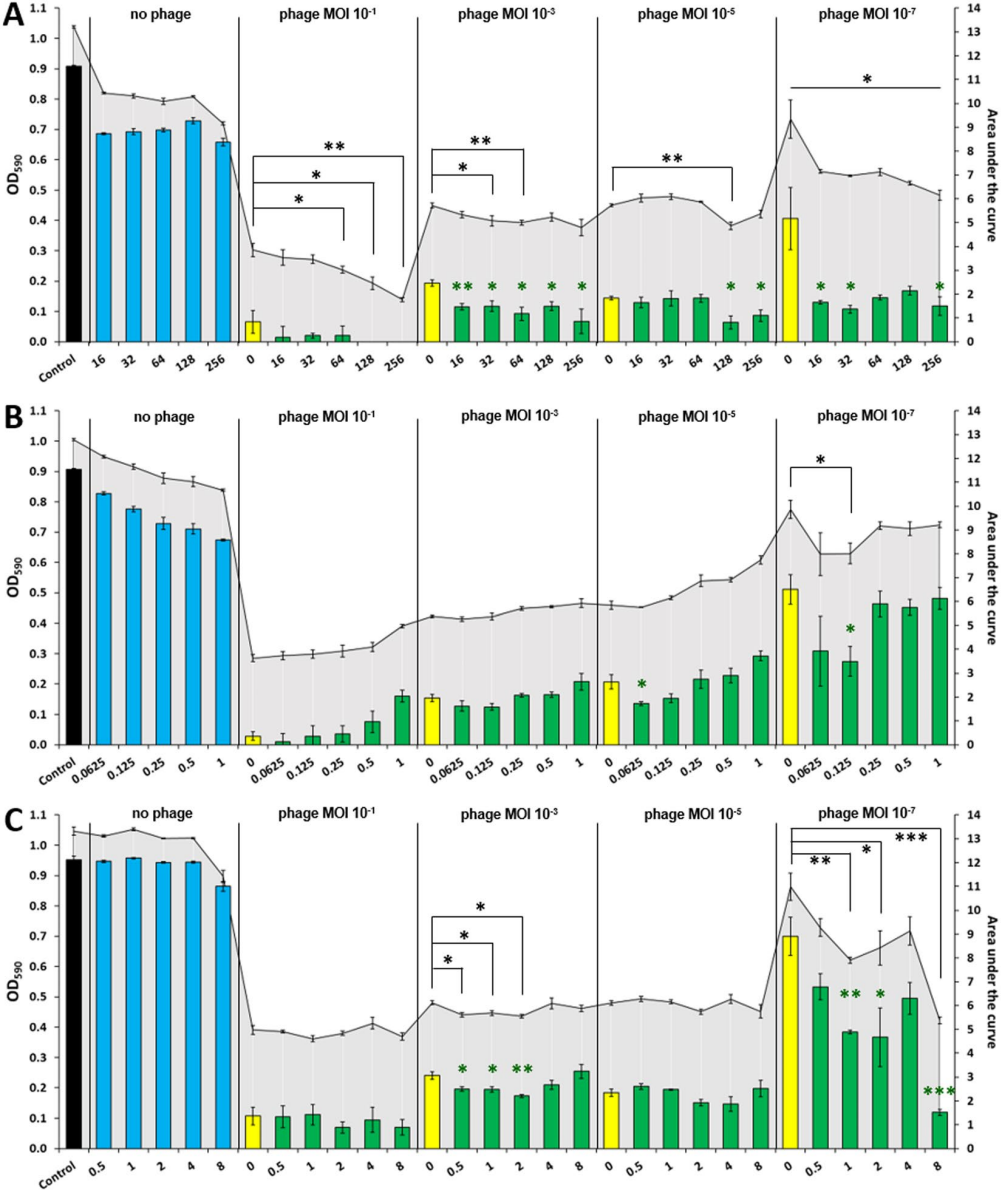
Combined infection assays against AB01. Reduction of multi-drug resistant *A*. *baumannii* strain AB01 in liquid medium is shown, using KARL-1 at four different MOIs and/or five different concentrations of each antibiotic; (**A**) meropenem, (**B**) ciprofloxacin, (**C**) colistin. The five antibiotic concentrations were in the range below and above the MICs determined for strain AB01. Reduction was measured via optical density at 590 nm (OD_590_). Bars indicate ODs measured after 16 h of treatment (endOD), with values given on the left y-axis (black bars: untreated bacteria, blue bars: antibiotic alone, yellow bars: phage alone, green bars: phage combined with antibiotic). The grey-scaled background displays the area under the curve (AUC) calculated for each 16h-treatment; values are given on the right y-axis. Numbers on the x-axis indicate the antibiotic concentrations (mg/l). Each experiment was performed in triplicate and the means ± standard errors are indicated. Stars indicate statistical support for an enhanced antibacterial effect of phage combined with antibiotics in comparison with the effect of phage alone at the same MOI; green stars refer to endOD, black stars to the AUC. ***p < 0.001, **p < 0.01, *p < 0.05.

Next, we tested the combined effect of KARL-1 and each of the three antibiotics, meropenem, ciprofloxacin, or colistin on strain AB01 (resulting growth/kill curves are displayed in Fig. S5). The evaluation considered two parameters, the OD reached at the end of the experimentation (end-OD), and the area under the curve (AUC), which reflects the average suppressive effect over the entire period of the 16 h infection assay. A stronger bacterial suppression using phage and antibiotics together was principally evident with both the end-OD and AUC, although the level of statistical significance was not equally well reached for both measures (Fig. 6). However, given the largely confirmative results, the following description therefore primarily refers to the end-OD. Each single antibiotic had no or only little antibacterial effect, irrespective of the dosage (blue bars in Fig. 6A–C). Conversely, the phage alone was effective at all tested MOIs, which ranged from 10^−1^ to 10^−7^, (yellow bars in Fig. 6A–C). The combined use of both, antibiotic and phage, revealed a dose dependent effect that differed for each tested antibiotic. When KARL-1 and meropenem were jointly applied at various mixing ratios, bacterial suppression was significantly increased for most combinations (green bars in Figs 6A and S5A,B). Bacteria were completely eradicated at an MOI of 10^−1^ and meropenem concentrations of 128 and 256 mg/l (verified by the lack of bacterial colonies after plating an aliquot on solid media after the 16 h of incubation). The addition of meropenem also moderately enhanced the persisting lytic effect of KARL-1 at the MOIs 10^−3^ and 10^−5^. However, the suppressive effect of both antibacterial agents together was most strongly pronounced when the phage was applied at an MOI of 10^−7^ (Figs 6A and S5B).

No significant improvement in bacterial treatment was observed when ciprofloxacin was applied together with KARL-1 except for two combinations: phage MOI 10^−5^/ciprofloxacin 0.0625 mg/l and phage MOI 10^−7^/ciprofloxacin 0.125 mg/l (Figs 6B and S5C).

Minor, but significant effects were seen with the combination of colistin and KARL-1 at an MOI of 10^−3^ (Fig. 6C). However, positive interactions were most strongly pronounced when the phage titer was lowest (i.e. MOI 10^−7^), and the colistin dosage highest, i.e. 8 mg/l colistin (Figs 6C and S5D).

**Figure 7:**
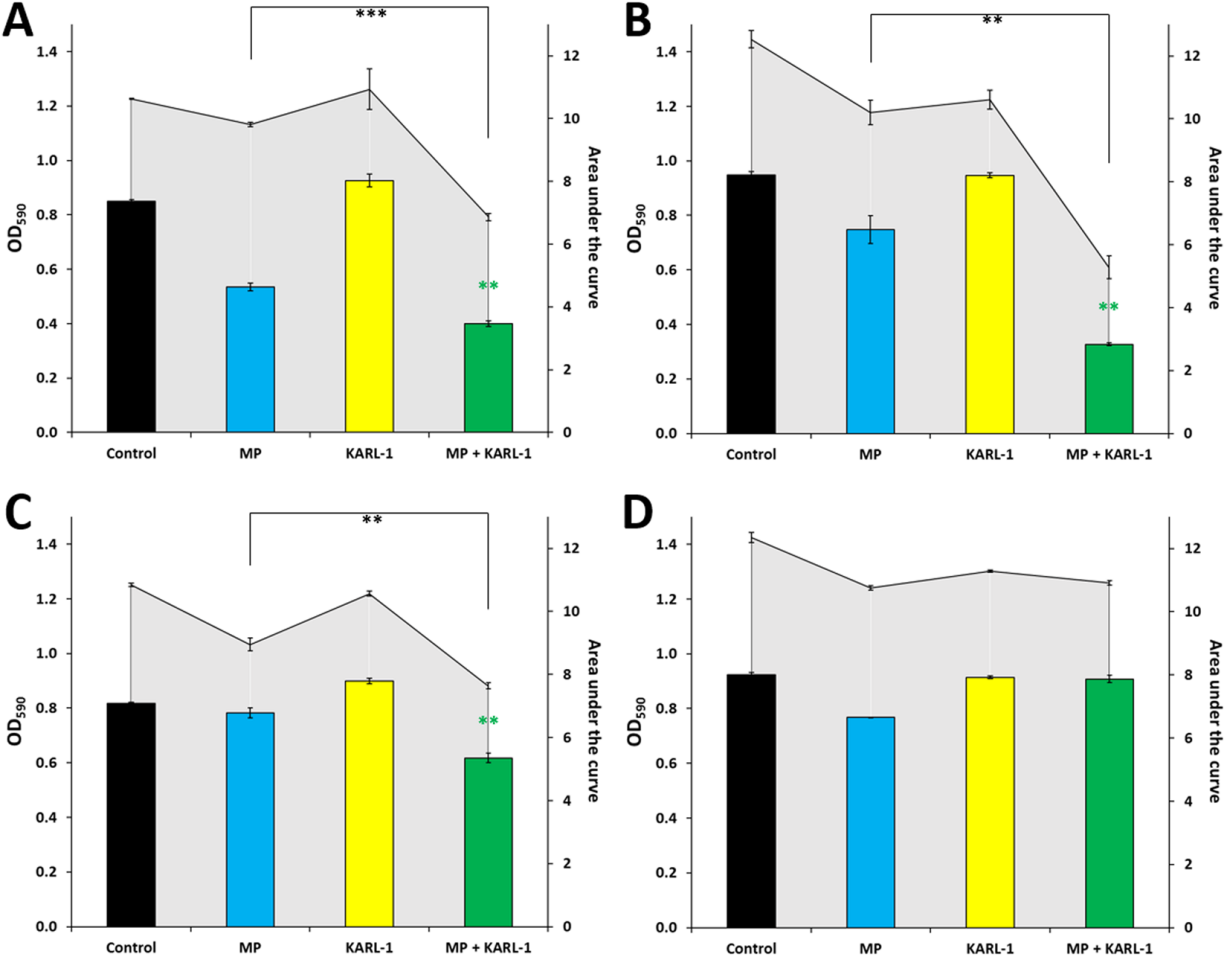
Combined infection assays against four additional strains. Reduction of four multi-drug resistant *A*. *baumannii* strains AB04 (**A**), AB16 (**B**), AB18 (**C**), and AB05 (**D**) in liquid medium is shown, using KARL-1 at a MOI 1 and meropenem (MP: 32 mg/l). Reduction was measured via optical density at 590 nm (OD_590_). Bars indicate ODs measured after 16 h of treatment (endOD), with values given on the left y-axis (black bars: untreated bacteria, blue bars: antibiotic alone, yellow bars: phage alone, green bars: phage combined with antibiotic). The grey-scaled background displays the area under the curve (AUC) calculated for each 16h-treatment; values are given on the right y-axis. Each experiment was performed in triplicate and the means ± standard errors are indicated. Stars indicate statistical support for an enhanced antibacterial effect of phage combined with antibiotics in comparison with the effect of the antibiotic alone; green stars refer to endOD, black stars to the AUC. ***p < 0.001, **p < 0.01, *p < 0.05.

Next, we tested the ability of meropenem to augment phage activity against four selected *A*. *baumannii* strains (i.e. AB04, AB05, AB16, and AB18), which were resistant against the highest number of antibiotics (Fig. S2). In three cases, application of KARL-1 alone initially led to a bacterial decline, however, regrowth occurred within a few hours. At the end of the experiment, phage-treated bacteria had returned to the same levels as the control (yellow bars, Fig. 7A–C). However, joined application of meropenem (32 mg/l) and phage (MOI 1) significantly reduced the three tenacious strains compared to the best acting antimicrobial alone, which was in this case meropenem (green versus blue bars, Fig. 7A–C). Lower dosages of meropenem and phage together did also show some improved effect compared to the single agents, which was, however, less strongly pronounced (data not shown). The positive interaction between meropenem and KARL-1 was not seen for strain AB05 (Fig. 7D). This strain was naturally unsusceptible to the phage as apparent from the initial investigations of the phage’s host range.

## Discussion

The vast majority of naturally occurring phages is still poorly explored^42–44^, and thus isolation of novel phage types or species and evaluation of their potential for phage therapy is a worthwhile endeavor. Of particular practical interest are lytic phages against opportunistic bacterial pathogens with multiple drug resistance, one of which is *A*. *baumannii*. This species has recently been classified by the World Health Organization as a “Priority 1 critical pathogen” for which research and development of new antibiotics is urgently needed^23^. Bioinformatic analysis has grouped all currently available *Acinetobacter* phage genomes into six discrete clusters along with two additional individual lineages represented by single phages^23^. KARL-1 groups within the phylogenetic radiation of “Cluster A”, the myovirus subfamily *Tevenvirinae* and is the first *Acinetobacter* phage reported from a German isolation source. It is also the second phage within this cluster that infects *A*. *baumannii* next to phage ZZ1^22^. Phages 133, Acj9, and Acj61 from that cluster use *Acinetobacter johnsonii* as host, while for phage Ac42 “*Acinetobacter* sp.” has been indicated as host^45,46^. Sharing the same bacterial species as host is reflected by the closer genetic relatedness of KARL-1 to ZZ1, although its phylogenetic position within Cluster A is clearly distinct (Fig. 3). In addition, the number of unique genes (singletons) within the genome of KARL-1 is of similar magnitude as the number of singletons in the genomes of the other five phages, all of which constitute different species^23,47^. Thus, KARL-1 may also be considered a novel phage species. To further evaluate the evolutionary relationship of those six phages, we performed separate phylogenetic analyses with the sequences of all 111 shared single-copy genes at protein level (Supplementary Table S1). Single-copy genes are of particular value for phylogenetic studies on prokaryote and eukaryote taxa^48–50^ and may as well provide important information about the phylogeny of phages. As expected, the clustering of the six phages varied from tree to tree, reflecting the individual evolutionary “history” of each of the 111 genes (data not shown). However, an inferred consensus tree (Fig. S6) matched well with the principle tree topology displayed in the dendrogram in Fig. 3. As a difference KARL-1, ZZ1, and Acj9 group more distinct from the phages Acj61, 133, and Ac42, the latter two of which now group tightly together (Fig. S6). This indicates a somewhat higher resolution power of the core gene analysis and suggests that the six phages diverged in evolutionary different time scales from a common ancestor (Fig. S6). It can be presumed that the characterization of additional phages affiliated with this cluster will help to gain better insights into the evolutionary relationships and biology of these phages^23^.

With a lytic spectrum of around 40% (inferred from 20 tested clinical isolates), KARL-1 can be considered to have a relatively broad host range. Although broader or comparable host spectra have been reported for other *Acinetobacter* phages (e.g. 68%, phage AP22^51^, 46%, ΒΦR1215 and ΒΦR2315^12^, 44%, Φkm18p^15^, 36%, BΦ-C62^52^), the host range of many *Acinetobacter* phages is quite narrow and lies somewhere between 2% and 30%^11,16,17,19,20,22,53^. In some studies, newly isolated phages were only able to infect their initial propagation strain^11,53^. In those cases, it is plausible to presume that the initial propagation strain, though susceptible to the phage, is not the actual host on which the respective phage preys in its natural environment.

While there is an ongoing discussion whether or not lytic phages are an alternative to antibiotics in order to combat multi-drug resistant bacteria^54^, a promising strategy appears to be the combined use of both antibacterial agents. Provided that there is no negative interference, the benefit of a simultaneous use can be two-fold: first, bacterial suppression may be stronger as a result of additive or synergistic effects^34^. Second, the bacterial pathogen is confronted with two different selective pressures, which may counteract or restrict the emergence of phage resistance during treatment^55,56^. In fact, cross-resistance between an antibiotic and phage seems less common than between two different types of antibiotics or phages^57^.

While the antibiotic meropenem alone had little effect on the propagation strain AB01, its combined use with KARL-1 increased bacterial suppression more or less intensively at all MOIs. However, the phage-supporting effect was most strongly pronounced with the lowest phage titer tested (i.e. MOI 10^−7^), irrespective of the meropenem dosage used in the infection assay (Fig. 6A). Meropenem is bactericidal and inhibits the cell wall synthesis of growing bacteria by irreversibly binding to essential penicillin-binding proteins, which catalyze the cross-linking of the peptidoglycan layer. Even though *A*. *baumannii* may have developed different resistance mechanisms such as carbapenemase-induced hydrolysis, porin protein alterations that result in reduced permeability of the outer membrane or increased efflux pump activity^58^, the presence of meropenem and phage together may lead to cellular stress responses, which might promote phage propagation in terms of larger burst sizes or shorter latent periods. Although in two cases ciprofloxacin and phage led to a minor, but significant suppressive effect, the overall data rather indicate a trend towards negative interactions between KARL-1 and ciprofloxacin. Ciprofloxacin inhibits the DNA gyrase as well as DNA topoisomerase IV, which are two bacterial enzymes essential for DNA replication and cell division^59^. T4-like phages (including KARL-1) also encode for a DNA topoisomerase, which might be essential for the initiation of phage DNA replication forks *in vivo*^60^. Thus, it is likely that ciprofloxacin might also inhibit those enzymes thereby suppressing phage replication. Even though negative interference between colistin and KARL-1 was not observed, the therapeutic improvement of their joined application was not substantial for most mixing ratios, except for the lowest phage dosage (i.e. MOI 10^−7^). In this case the simultaneous use of colistin is clearly eligible (Fig. 6C). Colistin binds to LPS with a subsequent destabilization of the outer membrane of gram-negative bacteria^61^, which could have affected positively phage adsorption and DNA injection.

Clearly more investigation is necessary to understand the precise mode of interactions between the antibiotics and phages at high and low doses. However, from our observations it can be concluded that comparably high dosages of KARL-1 as single agent are already fairly effective and a therapeutic improvement with an additional antibiotic is rather minor. Conversely, low amounts of the phage acting as sole agent are clearly less effective within a given time frame. But this lack of efficiency can in part be compensated or even be restored with simultaneous application of antibiotics (which is particularly true for meropenem and colistin, and – at least as a trend - even for ciprofloxacin). The positive interaction between antibiotics and low phage titer may have an important practical implication: during therapy an unknown fraction of phages may inevitably be eliminated or neutralized by the patient’s immune system^62,63^. Furthermore, there might be a poor penetration of administered phages towards their target bacteria and adsorption efficiency at site of infection could be low as well^64^. This means that under *in vivo* conditions only an unpredictable proportion of administered phages may actually be able to attack the bacterial pathogen, that is MOI_actual_ may be way below MOI_input_^64^. Because of these conceivable technical constraints, co-administration of supportive antibiotics could sustain a positive outcome of phage therapy, which otherwise could turn out to be insufficient or even fail in a timely manner, due to low phage densities at the site of infection. Notably, the beneficial effect was seen with different antibiotic concentrations varying over a range of factor 16, some of which reflect achievable dosing in human anti-infective therapy^65–68^. While we did not investigate the precise molecular mechanisms that drive the positive interactions between both antibacterial agents, we believe that such combinations can be extended to other antibiotics and phage types. It is therefore worthwhile to assess optimum combinations of different antibiotics and phage types with a particular emphasis on synergistic effects under conditions of a low phage-bacteria ratio in further studies.

The beneficial effect of a joined application of meropenem and KARL-1 was also apparent with the three strains AB04, AB16, and AB18. As single agent, KARL-1 caused an initial decline of bacteria after which regrowth started. The emergence of phage resistance was at least hampered with co-presence of meropenem, which again argues for the combined use of phage and antibiotics as the preferable therapeutic rationale. However, the strain AB05, which was a priori non-susceptible to phage infection, remained tenacious even when meropenem was co-added. At this point it remains unclear whether a therapeutic improvement can only be expected within the radiation of the phage’s natural host range. Further studies are needed in which phage-resistant strains are challenged with phage/antibiotic combinations to find clear answers.

In conclusion, KARL-1 represents an interesting new candidate for therapy against multi-drug resistant *A*. *baumannii* and the combined use with an antibiotic (e.g. a carbapenem, which as a single agent has become ineffective) may considerably improve the outcome. This antibacterial effect can probably be optimized by modulating the time points at which either agent is applied, as shown recently with *P*. *aeruginosa*^34^. The whole repertoire of conceivable combinations of antibiotics and phage types has by far not been sufficiently explored yet, which is true for *A*. *baumannii*, but also for other multi-drug resistant bacteria. Further studies are therefore warranted to achieve ever more promising results with phages against pathogenic bacteria that have become insensitive to antibiotics.

## Methods

### Bacterial strains

In total, 21 multi-drug resistant *A*. *baumannii* strains (AB01-AB21) isolated from various clinical specimen at the University Hospital RWTH Aachen, Germany, were initially selected for this study. Species identity was verified via MALDI-TOF mass spectrometry (Microflex LT, Bruker Daltonik GmbH, Bremen) for all strains except for AB17, which was disregarded for further studies. Unless otherwise stated, bacterial strains were generally grown in Lysogeny Broth-LB (NaCl 1% w/v, tryptone 1% w/v, yeast extract 0.5% w/v). The automated antimicrobial susceptibility testing of clinical isolates was performed using the VITEK2 system (bioMérieux, Marcy-l′Étoile, France). The identification of carbapenem-resistance genes was performed by polymerase chain reaction (PCR) using the Xpert Carba-R kit (Cepheid Inc., USA)^69^. All but one strain were identified as potential producers of one of the following carbapenemases: NDM, IMP-1, IMP1 + OXA-58, OXA-23, OXA-24, or GIM (Fig. S2). Bacterial strains were further differentiated via enterobacterial repetitive intergenic consensus PCR (ERIC-PCR) as described previously^70,71^. Briefly, ERIC-PCR was performed in a 50 µl volume and each reaction mixture contained 2.5 U of GoTaq G2 Flexi DNA Polymerase supplied with GoTaq Flexi Buffer Complete: (Promega, Madison, WI, USA), 4 mM MgCl_2_, 0.2 mM dNTPs (Roche Applied Science, Penzberg, Germany), 2.0 µM of each primer, and approx. 50 ng of template DNA. Genomic DNA from pure cultures was obtained using the QIAamp DNA Mini Kit (Qiagen, Hilden, Germany) according to the guidelines of the manufacturer. The concentration of each purified genomic DNA sample was measured with the NanoVue Plus (GE Healthcare, Little Chalfont, UK). The primers for ERIC-PCR were: “ERIC-1” 5′-ATGTAAGCTCCTGGGGATTCAC-3′ and “ERIC-2” 5′-AAGTAAGTGACTGGGGTGAGCG-3′. The thermal profile of the PCR reaction started with an initial denaturation at 95 °C for 7 min, followed by 35 cycles of denaturation at 94 °C for 30 s, primer annealing at 52 °C for 1 minute and extension at 72 °C for 8 min, and one cycle of further extension at 72 °C for 16 min. The DNA-fragments generated via ERIC-PCR were run on a 1.5% TBE-agarose gel at 80 V for 3 h at room temperature and visualized using the GelStudio SA System (Analytik Jena, Jena). DNA fingerprint data were analyzed via GelQuest and ClusterVis (http://www.sequentix.de/gelquest/help/index.html). For calculation of distance matrices the Jaccard-index and for calculation of cluster trees the neighbor joining algorithm was used.

### Phage preparation and storage

KARL-1 was initially obtained from an environmental sample using strain AB01 as propagation host^41^. Phages were purified by successive single plaque isolation and propagation. In order to obtain high titer phage lysates phages were mixed with a mid-log-phase of the propagation strain AB01 and shaken at 200 rpm overnight at 37 °C. After overnight incubation, the host-phage suspension was centrifuged at 2,330 × g for 10 min and filtered twice with a 0.45-µm-pore-size and a 0.2-µm-pore-size sterile filter. Phage titer was determined as the number of plaque forming units (pfu/ml) by the Double Agar Overlay Plaque Assay as described previously^72^. For short-term storage (e.g. several weeks) phage lysates were stored at 4 °C. For long-term storage phages were mixed with glycerol (20% (v/v)) in equal parts and stored in CryoPure Tubes (Sarstedt, Nuembrecht, Germany) at −196 °C in liquid nitrogen.

### Optimal temperature for phage-induced antibacterial activity

To assess the optimal temperature for phage-induced cell lysis, KARL-1 was incubated with its propagation strain AB01 by serial dilution spot testing at different temperatures, (i.e. RT, 30 °C, 35 °C, 37 °C, 40 °C, 42 °C, and 45 °C). The optimal temperature for antibacterial activity was determined by comparing the minimum phage concentration required to form an inhibition zone in the bacterial lawn^22^.

### Host range

The host range was determined for the 20 multi-drug resistant clinical strains *A*. *baumannii* AB01-AB21 and for seven reference strains: 1. *A*. *baumannii* strain “10322” (DSM-no. 24110), 2. *A*. *baumannii* type strain “2208” (ATCC 19606, DSM-no. 30007), 3. *A*. *baumannii* strain “Bouvet and Grimont 8” (ATCC 17978, DSM-no. 105126), 4. *A*. *pittii* type strain “Nemec” (ATCC 19004, DSM-no. 25618), 5. *A*. *nosocomialis* type strain “RUH 2376” (DSM-no 102856), 6. *A*. *calcoaceticus* type strain “Delft L360” (ATCC 23055, DSM-no. 30006), 7. *A*. *calcoaceticus* “SW1” (DSM-no. 16962). All reference strains were purchased from the German Collection of Microorganisms and Cell Cultures DSMZ (https://www.dsmz.de/home.html). Strains were cultivated in 5 mL LB medium overnight at 37 °C and 200 rpm. After adjustment to an OD_600_ of 1, 50 µl of the culture were plated on solid LB media and 10 µL of the phage at various dilutions (range from 10^8^ pfu/ml to 10^2^ pfu/ml) spotted onto the plate. Productive lysis was assumed in cases where individual plaques were visible within the spotting zone at appropriate phage dilutions.

### Determination of burst size and phage adsorption profile

A one-step growth curve was performed using the initial propagation strain AB01 as described previously^72^. Briefly, 900 µL of bacterial culture (with an OD at 600 nm of 0.4, equal to approximately 10^8^ cfu/ml) was mixed with 100 µL of phage suspension (10^7^ pfu/ml) to obtain a multiplicity of infection of 0.01. Phages were allowed to adsorb for 10 min at 37 °C, after which the mixture was diluted to 10^−4^. Triplicate samples taken at 5 min intervals for 30 min and then at 10 min intervals for 90 min were mixed with bacterial cultures and plated in order to obtain countable plaques in the bacterial lawn after overnight incubation. Phage titers obtained at begin and end of the experiment were used to estimate the burst size.

For determination of the adsorption profile, 19 ml of bacterial culture (strain AB01, approximately 10^7^ cfu/ml) were mixed with 1 ml phage suspension (10^5^ pfu/ml) to obtain a multiplicity of infection of 5 × 10^−4^ and incubated at 37 °C and 200 rpm. Duplicate samples were taken every 3 min and filtered with 0.2-µm-pore-size sterile filter. Each filtrate was mixed undiluted and diluted by 10^−1^ with bacterial culture, and plated in order to obtain countable plaques in the bacterial lawn after overnight incubation. The number of free phage particles per filtrate was used to establish the adsorption profile.

### Transmission electron microscopy (TEM)

High-titer phage lysate was exchanged in HEPES buffer via Amicon Ultra-0.5 mL Centrifugal Filter Units 100 K (Merck, Darmstadt, Germany). Phages were allowed to adsorb on glow discharged formvar-carbon-coated nickel grids (Maxtaform, 200 mesh, Plano, Wetzlar, Germany) for 10 min. Samples on grids were stained by placing on a drop of 1% phosphotungstic acid (in aqua dest., adjusted to pH 7.2; Agar Scientific Ltd., Stansted, United Kingdom). After air drying, samples were examined using a TEM LEO 906 (Carl Zeiss, Oberkochen, Germany), operating at an acceleration voltage of 60 kV.

Wide-angle Dual Speed 2K-CCD-Camera 14 bit (Tröndle, TRS Moorenweis, Germany) and analysis software IMAGE SP Professional (SISPROG, Tröndle, Moorenweis, Germany) were used to photograph observations.

Description of phage morphology and determination of the size of phage head and tails were performed based on five selected virions displayed on the electron micrographs (average size ± standard deviation is given).

### Phage genome sequencing and *in silico* genome analysis

Whole genome sequencing was performed by use of the MiSeq platform (Illumina, San Diego, U.S.) according to the product manual (Nextera XT DNA Sample Preparation Guide [version October 2012]). Prior to the extraction of phage DNA, remnants of bacterial DNA were removed through a DNAse digest for 15 min. Phage genomic DNA extraction was performed using the QIAamp DNA Mini Kit (Qiagen, Hilden, Germany) according to the product manual (DNA Purification from Blood and Body Fluids [Spin Protocol]). For sequencing, a paired-end library was generated using the Nextera XT Library Prep Kit, and 2 × 150 bp reads were generated using the MiSeq v2 Reagent Kit. *De novo* assembly of reads was performed via the St. Petersburg genome assembler (SPAdes)^73^. Via genome wide BLASTn analysis the most closely related phages deposited in GenBank were searched and the result visualized via BRIG^74^. Potential ORFs were identified using GeneMark.S^75^ and annotation was performed using PHASTER^76^. Putative tRNA genes were identified using the tRNAscan-SE program^77^. Translated ORFs were further compared with the five most closely related phages ZZ1, Acj9, Acj61, Ac42, and 133, all belonging to the subfamily *Tevenvirinae* (“Cluster A”)^23^, via BLASTx and results were manually sorted according to the degree of sequence identity. The genome nucleotide sequence of KARL-1 has been deposited at GenBank (accession number: MH713599).

### Phylogenetic analysis

Comparative analysis of shared and non-shared orthologous genes was performed in order to elucidate the phylogenetic relationship of KARL-1 with other recognized *Acinetobacter* phages. To this end, translated ORF sequences were clustered into orthologous proteins using OrthoVenn with an e-value of 1e^-^5 and an inflation value of 1.5^50^. OrthoVenn is based on the heuristic approach OrthoMCL to identify ortholog groups^78^. Pairwise comparison of shared orthologs was performed with eleven phage genomes as representatives of the recognized *Acinetobacter* phage clusters A – F^23^. Results were transformed into a binary matrix indicating the presence/absence of each gene for all pairs of phages. This matrix was used for calculating Jaccard distances for each phage pair. Phylogenetic tree reconstruction with the neighbor-joining algorithm was performed with the computer software MEGA 6.06 based on the calculated Jaccard-distances.

For further analyzing the relationship of KARL-1 with the other five members of the *Tevenvirinae* (Cluster A), the total number of genes, number of shared genes, and number of singletons was determined using OrthoVenn^50^. From the set of 111 single-copy core genes identified in all six phages separate phylogenetic tree reconstruction was performed based on the amino-acid sequences using the treeing algorithms implemented in OrthoVenn. For all 111 resulting phylogenetic trees the frequency with which each phage grouped together with one of the other phages was determined and the counts were used for creation of a data matrix. From this data matrix a consensus tree was calculated with the neighbor-joining algorithm (MEGA 6.06).

### Infection of planktonic cells

Initial infection assays were performed with the propagation strain AB01 and with the two strains AB02 and AB03. The latter two strains were selected for comparison, because previous efforts with those two strains to propagate phages from the same environmental sample were unsuccessful.

Infection of planktonic cells was done at the exponential phase of bacterial growth. To this end, an overnight pre-inoculum was used to inoculate 10 ml of fresh 2x LB-medium to allow growth to approximately 5 × 10^8^ cfu/ml. In all experiments, 100 µl of a host suspension were mixed with 100 µl of the phage (at four different concentrations) in order to obtain four MOIs. Control experiments were performed with 2x LB-medium mixed with an equal volume of phage buffer. The infection of planktonic cells was performed in 96-well microtiter plates. The microtiter plates were sealed with an adhesive tape, subsequently. With the aid of a sterile syringe, holes were made at the edge above every well to ensure ventilation and supply of oxygen. The microtiter plates were placed into the microplate reader SpectraMax i3 (Molecular Devices, Sunnyvale, U.S.) and incubated for 16 h at 37 °C while horizontally shaking (system modus: moderate). The OD_590_ was measured at time intervals of 20 min for the entire duration of the experiment. All experiments were performed in triplicate. The OD_590_ data were used for calculating the area under the curve (AUC) via numerical integration with the formula

$${\sum }_{i=0}^{48}\frac{f(i\ast {\rm{\Delta }}t)+f(i+1\ast {\rm{\Delta }}t)}{2}\ast {\rm{\Delta }}t,$$

*with* ${\rm{\Delta }}t=20\,min\,=0.33\,h$, *and* $f(i\ast {\rm{\Delta }}t)$ representing the OD_590_-values measured every 20 min for 16 h.

The three antibiotics meropenem, ciprofloxacin, and colistin (Sigma-Aldrich, St. Louis, U.S.) were tested in combination with KARL-1 in order to assess positive interactions during antibacterial treatment of strain AB01. Four different phage concentrations (i.e. MOIs 10^−1^, 10^−3^, 10^−5^, and 10^−7^) were combined with five different antibiotic concentrations (i.e. meropenem: 16 to 256 mg/l, ciprofloxacin: 0.0625 to 1 mg/l, colistin: 0.5 to 8 mg/l. The five antibiotic concentrations were in the range below and above the MICs determined for strain AB01 (i.e., meropenem ≥ 32 mg/l, ciprofloxacin 0.09 mg/l, colistin 2 mg/l) and largely reflected achievable dosing in human anti-infective therapy^65–68^.

In order to verify the informative value of the optical density data, a representative set of samples were plated after the 16h-experiments. Resulting cell counts reflected well the OD-measurements. In particular, reduction of OD due to the presence of an antibiotic was correlated with reduction of bacterial cells and not with altered cell morphology (data not shown).

We also tested the effect of meropenem and KARL-1 against four additional clinical *A*. *baumannii* strains AB04, AB05, AB16, and AB18. These strains were selected as they were among those with resistance against the highest numbers of antibiotics, Fig. S2. For all four strains the MICs determined for meropenem were ≥32 mg/l.

### Statistical analysis

In this study, we were interested to see if the phage + antibiotic combination had a stronger antibacterial effect than the best acting antimicrobial alone with the same dosage. The null hypothesis of no difference between the two treatments was tested with a one-tailed t-test, with a significance level of P < 0.05.

## Electronic supplementary material

Supplementary Figures

Dataset 1
