# Supplementary material for: Enhanced antibacterial effect of the novel T4-like bacteriophage KARL-1 in combination with antibiotics against multi-drug resistant Acinetobacter baumannii
Source: Sci Rep. 2018 Sep 20;8:14140. doi: 10.1038/s41598-018-32344-y (PMC6147977; doi:10.1038/s41598-018-32344-y)
Supplement: Supplementary file 1 — Supplementary Figures [file 41598_2018_32344_MOESM1_ESM.pdf]

Enhanced antibacterial effect of the novel T4-like bacteriophage KARL-1 in combination with antibiotics against multi-drug resistant *Acinetobacter baumannii*

Mathias Jansen[1*], Adam Wahida[1,2*], Simone Latz[1], Alex Krüttgen[1], Helga Häfner[3], Eva Miriam Buhl[4], Klaus Ritter[1], Hans-Peter Horz[1]

[1]Institute of Medical Microbiology, RWTH Aachen University Hospital, Aachen, Germany
[2]Third Medical Department for Haematology and Oncology, Klinikum rechts der Isar, Technical University of Munich, 81675 Munich, Germany
[3]Department of Infection Control and Infectious Diseases, RWTH Aachen University Hospital, Aachen, Germany
[4]Electron Microscopy Facility, RWTH Aachen University Hospital, Aachen, Germany
*First Co-authorship

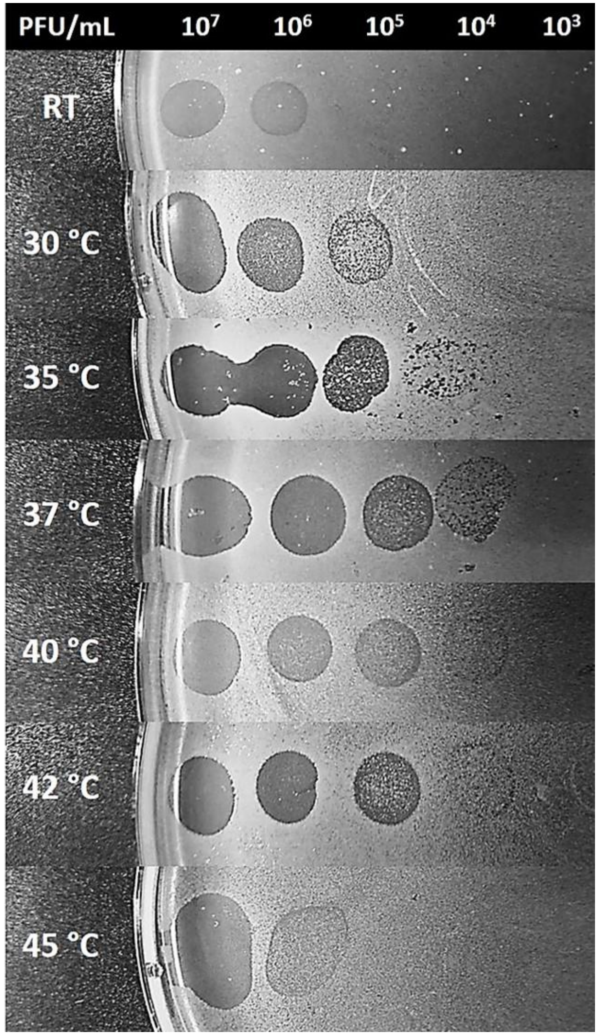

Fig. S1 Lysis activity of phage KARL-1 at various concentrations and temperatures against its initial propagation strain AB01.

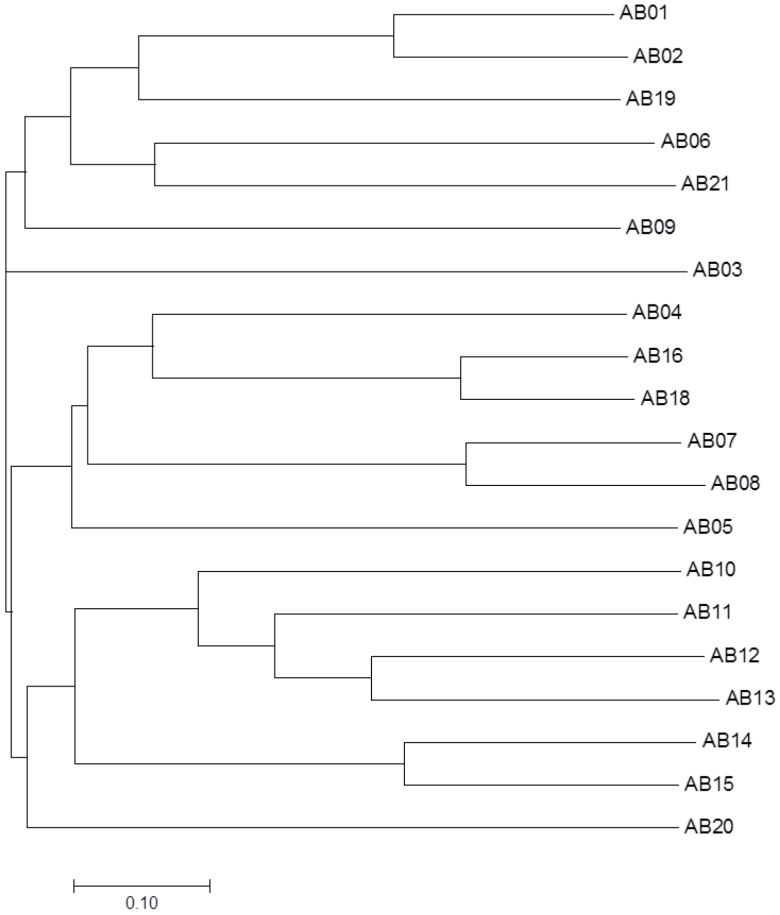

Strain	Phage Susceptibility	Carbapenems		Fluoroquinolone	Aminoglycosides		Diamino-pyrimidine/ Sulfonamide	Polymyxin	Carbapenemase
		IMP	MPM	CI	GM	TM	TMP/SMX	COL	
AB01	++	R	R	S	S	S	S	S	NDM
AB02	++	R	S	S	S	S	S	S	NDM
AB19	-	I	R	S	S	S	R	S	IMP-1 + OXA-58
AB06	++	R	S	S	S	S	S	S	NDM
AB21	++	S	I	S	S	S	S	S	NDM
AB09	-	R	R	R	R	S	R	S	OXA-23
AB03	-	I	R	S	S	S	R	S	GIM
AB04	++	R	R	R	R	R	S	S	OXA-23
AB16	++	R	R	R	R	R	S	S	neg.
AB18	++	R	R	R	R	R	R	S	OXA-23
AB07	-	R	R	S	S	n.d.	S	n.d.	NDM
AB08	++	R	R	S	S	S	S	S	NDM
AB05	+	R	R	R	R	S	R	S	OXA-24
AB10	+	R	R	S	S	S	R	S	IMP-1 + OXA-58
AB11	+	R	R	S	S	S	R	S	IMP-1 + OXA-58
AB12	+	R	R	S	S	n.d.	R	n.d.	IMP-1
AB13	+	R	R	S	S	S	R	S	IMP-1 + OXA-58
AB14	+	I	R	S	S	S	R	S	IMP-1
AB15	+	I	R	S	S	S	S	S	IMP-1
AB20	+	I	R	S	S	R	R	S	IMP-1 + OXA-58

Fig. S2 Neighbor-joining dendrogram resulting from ERIC-PCR for multi-drug resistant *A. baumannii* clinical isolates (AB01-21), resistance profiles, and identified carbapenemases. IMP: Imipenem; MPM: Meropenem; CI: Ciprofloxacin; GM: Gentamicin; TM: Tobramycin; TMP/SMX: Trimethoprim/Sulfamethoxazole; COL: Colistin; R = resistant, I = intermediate, S = sensitive, n.d.: not determined; ++ = Productive lysis by phage KARL-1; + = Lysis from without; - = strain not susceptible to phage KARL-1

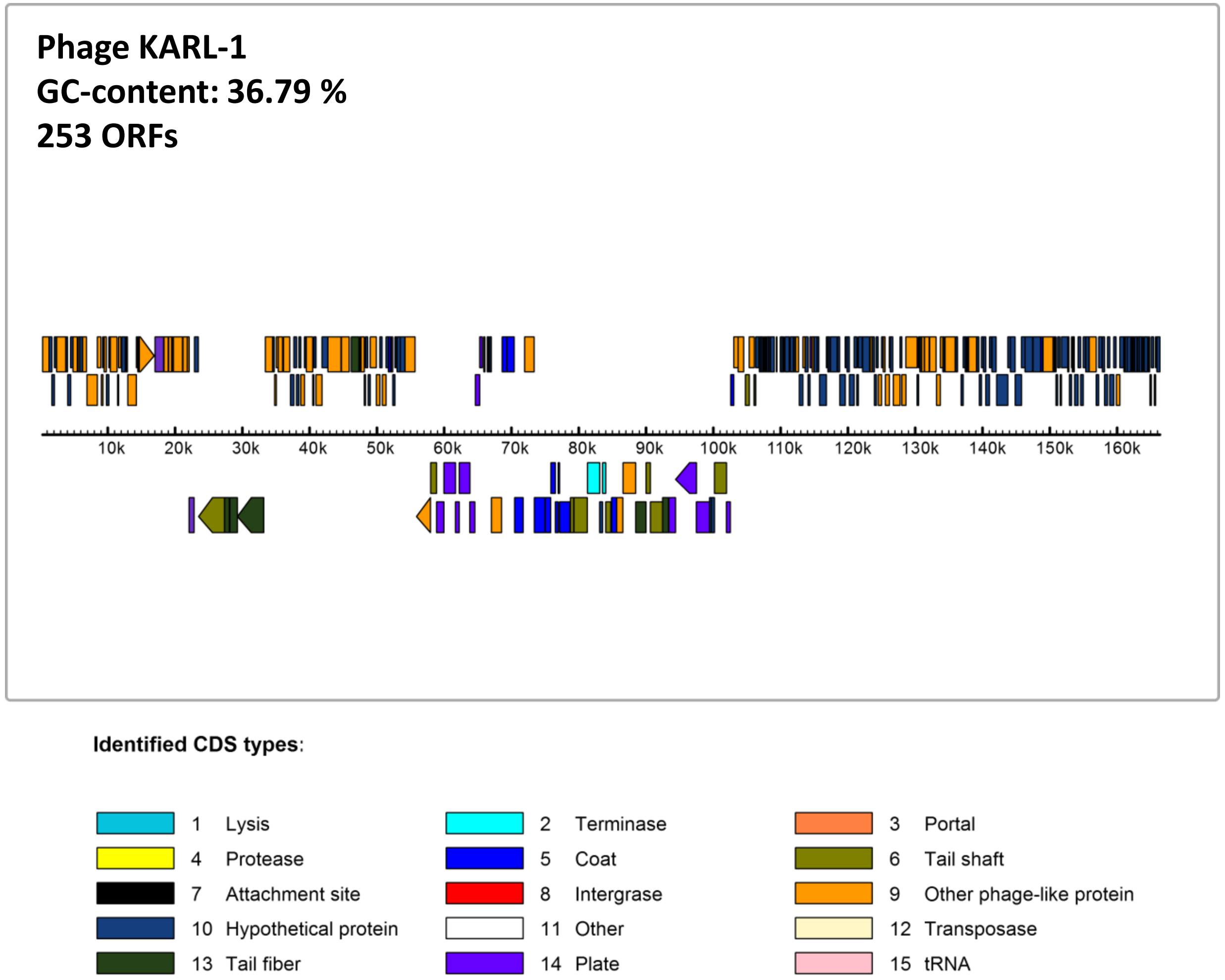

Fig. S3 Gene annotation and orientation of phage KARL-1 via PHAST. For a detailed listing, see Table S1.

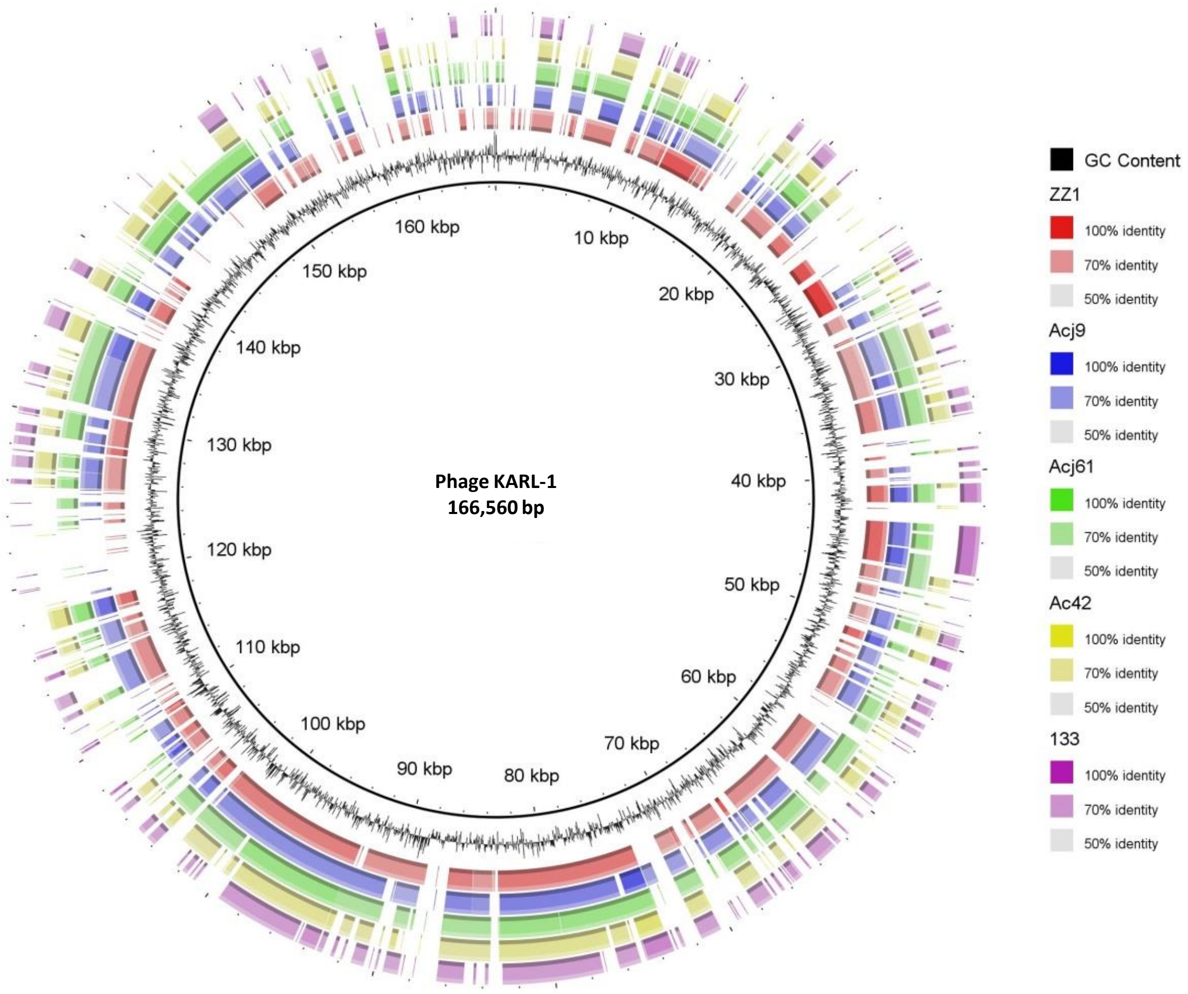

Fig. S4 Visualization of the BLASTn results using BRIG. Comparison of phage KARL-1 with five members of the subfamily *Tevenvirinae* (Cluster A), i.e. phages ZZ1, Acj9, Acj61, AC42, and 133.

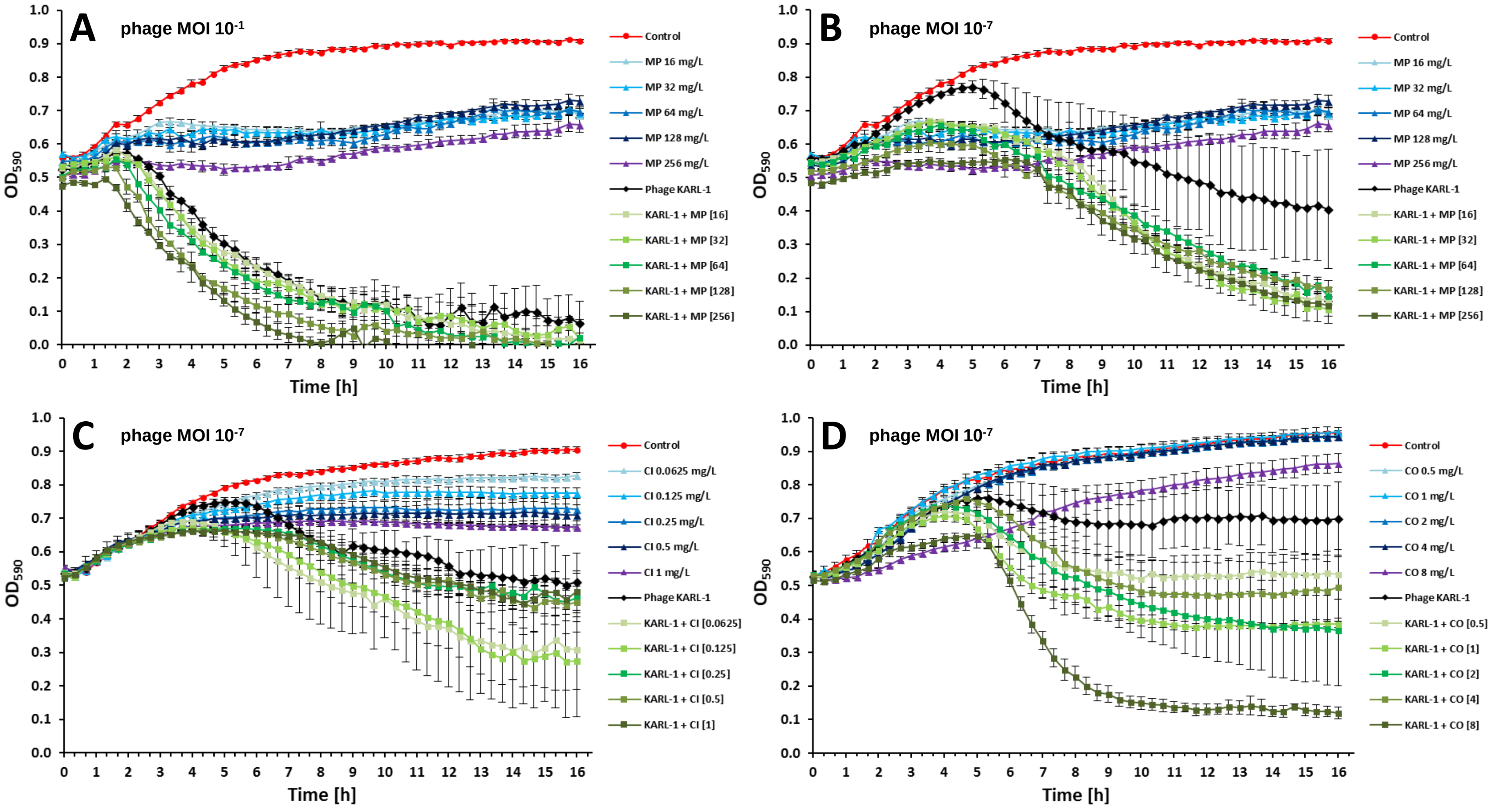

Fig. S5 Activity of phage KARL-1 against planktonic cells of multi-drug resistant *A. baumannii* strain AB01 with and without antibiotics; (A) phage MOI 10^{-1} and meropenem (MP) ranging from 16 to 256 mg/l; (B) phage MOI 10^{-7} and meropenem (MP) ranging from 16 to 256 mg/l; (C) phage MOI 10^{-7} and ciprofloxacin (CI) ranging from 0.0625 to 1 mg/l; (D) phage MOI 10^{-7} and colistin (CO) ranging from 0.5 to 8 mg/l; Bacterial reduction was measured via optical density at 590 nm (OD_{590}). Each experiment was performed in triplicate, bars indicate standard deviation.

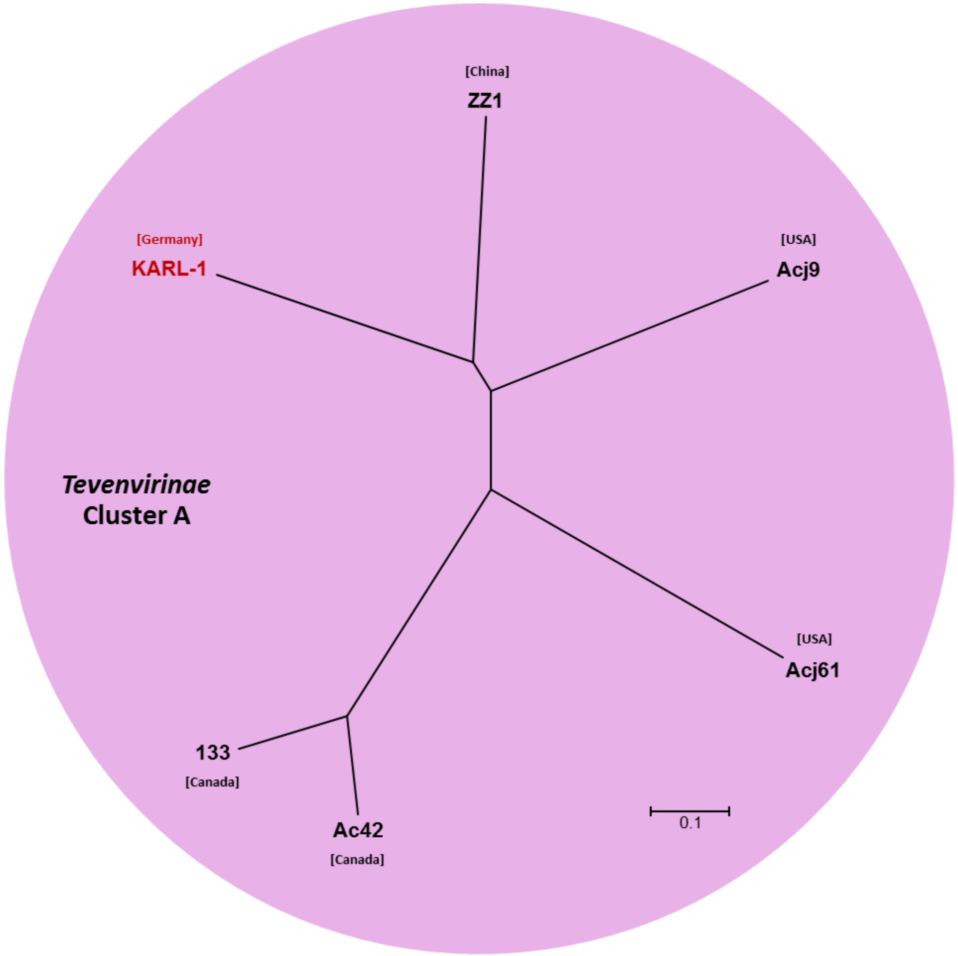

Fig. S6 Distance dendrogram showing the genetic relatedness of phage KARL-1 within the subfamily *Tevenvirinae* (Cluster A). The dendrogram represents a consensus tree based on phylogenetic reconstruction using the 111 single-copy genes present in all six phages (core genes); The node labels represent individual phage genomes and their country of isolation. The accession numbers of the phage genomes are provided in Table 1 of the main manuscript. The scale bar represents the number of genes (reciprocal in percent) that support the grouping of any two phages in the tree topologies (for further details see the Methods-section in the main manuscript).
